# Guide for Selecting Experimental Models to Study Dietary Fat Absorption

**DOI:** 10.3390/nu17233644

**Published:** 2025-11-21

**Authors:** Andromeda M. Nauli, Ann Phan, Karen Mai, Kathleen Tran, Surya M. Nauli

**Affiliations:** 1 Department of Biomedical Sciences, Western Michigan University Homer Stryker M.D. School of Medicine, Kalamazoo, MI 49008, USA; 2 Desert Valley Hospital, Victorville, CA 92395, USA; 3 College of Pharmacy, Marshall B. Ketchum University, Fullerton, CA 92831, USA; 4 Department of Biomedical and Pharmaceutical Sciences, Chapman University, Irvine, CA 92618, USA; nauli@chapman.edu; 5 Department of Medicine, University of California, Irvine, Irvine, CA 92697, USA

**Keywords:** intestinal lipid absorption, dietary fat digestion, chylomicron assay, intestinal VLDL, apolipoprotein, lymph, enterocytes, intestinal epithelial, gastrointestinal, fat malabsorption

## Abstract

Dietary fat absorption is a complex, multi-step process involving digestion, enterocyte uptake, intracellular trafficking, re-esterification, and transport via lipoproteins into circulation. Because dietary fat absorption plays a central role in lipid homeostasis, metabolic syndrome, and fat malabsorption disorders, its study has a broad biomedical significance. However, experimental investigation of this process is technically challenging due to the short lifespan of enterocytes, the dual lymphatic and portal transport routes, and the need to trace the metabolic fate of absorbed lipids. This review summarizes and critically evaluates the major experimental systems used to study dietary fat absorption, highlighting their respective strengths, limitations, and utility. A guide for selecting the most appropriate model to study specific stages of dietary fat absorption is also presented. Ultimately, because each model carries inherent methodological constraints, integrative experimental strategies that combine complementary will be necessary to link mechanistic insights with physiological relevance.

## 1. Introduction

Dietary fat absorption involves four major stages, namely digestion, absorption, intracellular processing, and transport [1]. Ingested lipids, which consist primarily of triglycerides, phospholipids, and cholesterol, first undergo digestion, a process that relies heavily on bile acids and pancreatic enzymes. Once digested, the resulting fatty acids, monoglycerides, lysophospholipids, and cholesterol are absorbed by enterocytes, a step often referred to as uptake [2]. Of note, cholesteryl esters need to be hydrolyzed into cholesterol prior to their uptake.

**Figure 1:**
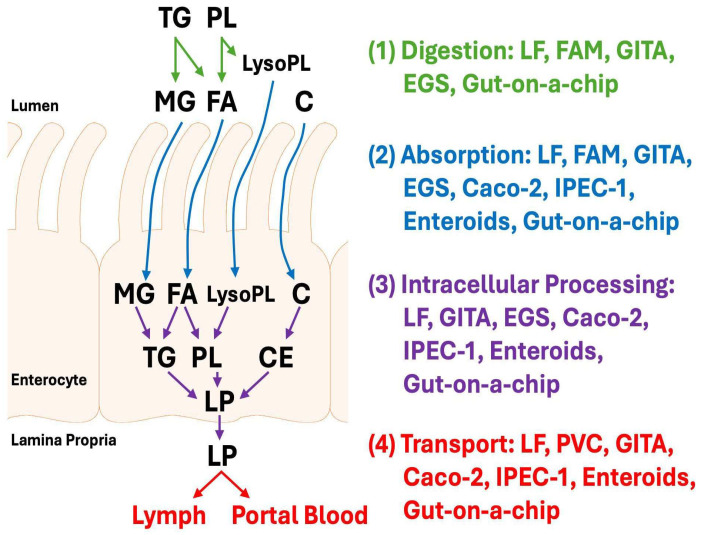
Dietary fat absorption proceeds through four major stages: (1) digestion, (2) absorption, (3) intracellular processing, and (4) transport. Stage 1 (green): Digestion occurs in the intestinal lumen and depends on bile acids and pancreatic enzymes, which hydrolyze triglycerides (TG) into monoglycerides (MG) and fatty acids (FA), and phospholipids (PL) into lysophospholipids (lysoPL) and FA. Stage 2 (blue)**:** These lipid digestion products, including cholesterol (C), are then taken up by enterocytes during absorption. Stage 3 (purple): Inside the enterocyte, they are re-esterified—cholesterol primarily to cholesteryl ester (CE)—and assembled into intestinal lipoproteins (LP), which are secreted by exocytosis into the lamina propria. Stage 4 (red): While most LP are transported via the lymphatic circulation, smaller LP can bypass the lymphatics and enter the portal blood instead. Experimental models: The lymph fistula (LF), gavage followed by intestinal tissue analysis (GITA), and gut-on-a chip models can be used to examine all four stages of dietary fat absorption. The fecal analysis method (FAM) is primarily suited for assessing absorption, though it can also yield insights into digestion. The everted gut sac (EGS) model is used for studying digestion, absorption, and intracellular processing, but is suboptimal for transport studies due to incomplete LP recovery and the inability to collect lymph or portal blood samples. Caco-2, IPEC-1, and enteroid models allow investigation of absorption, intracellular processing, and transport, though they are not ideal for digestion studies. Finally, the portal vein cannulation (PVC) and human fetal gut models are mainly used to study portal transport and development aspect of dietary fat absorption, respectively. For the transport-related studies, both LF and PVC models provide incomplete LP recovery, and in vitro models (Caco-2, IPEC-1, enteroids, and gut-on-a-chip) do not fully recapitulate physiological conditions. Through analyzing the intestinal tissues with transmission electron microscope, GITA can also serve a complementary model to study transport.

Once inside the enterocytes, these lipids undergo intracellular processing, which includes trafficking, re-esterification, and packaging into lipoproteins [3]. These lipoproteins are then released into the lamina propria. Finally, transport occurs as the lipoproteins enter the lymphatic circulation. However, some smaller lipoproteins, such as small very low-density lipoproteins (VLDLs), can bypass the lymphatics and enter the portal blood directly [4]. These four stages of dietary fat absorption, including the experimental models that can be used to study them, are illustrated in Figure 1.

The study of dietary fat absorption has become increasingly important due to its relevance to metabolic health. The small intestine, through its regulation of dietary fat digestion, absorption, and transport, plays a key role in determining the pathogenesis of metabolic syndrome in general, and the development of abdominal visceral fat in particular [5]. As the primary organ responsible for these processes, the small intestine influences circulating triglyceride levels and determines the sites of lipid deposition—both key factors in metabolic syndrome [6].

This regulatory capacity is partly mediated by the size of intestinal lipoproteins. Based on our findings [7,8] and those of others [9,10], smaller intestinal lipoproteins can enter the portal circulation because the endothelial gaps in blood capillaries are narrow, whereas larger lipoproteins pass into the lymphatic system, which has wider endothelial gaps. The distinct transport routes of these lipoproteins likely influence the eventual deposition of dietary lipids. Larger lipoproteins, retained longer within the intestinal wall due to their size and the low pressure of the lymphatic system [11], may have triglycerides that are more prone to local hydrolysis and preferentially taken up by surrounding abdominal visceral fat. In contrast, smaller lipoproteins that enter the portal vein are rapidly delivered into the general circulation, with minimal retention in the intestinal wall due to the higher pressure of the blood circulation. Consequently, the triglycerides they carry are less likely to be taken up by abdominal visceral fat compared with those transported in larger lipoproteins via the lymphatic system. Consistent with this concept, previous studies have shown that impaired lymphatic function—and the resulting retention of lipoproteins within the abdominal wall—is associated with the accumulation of abdominal visceral fat [12,13].

Accordingly, selecting appropriate experimental models of dietary fat absorption—especially those that capture the transport phase, is crucial for advancing our understanding of how intestinal lipid handling contributes to the development of metabolic syndrome.

Metabolic syndrome has become alarmingly prevalent in modern society. In the United States, approximately one-third of adults and nearly half of individuals over 65 years of age are affected [14]. Without effective interventions, these numbers are expected to continue rising.

Conversely, when dietary fat absorption is impaired, fat malabsorption ensues [15]. This condition is characterized by steatorrhea (fatty stools), unintentional weight loss, and potential deficiencies of fat-soluble vitamins (A, D, E, K). Fat malabsorption can result from a variety of underlying conditions, including exocrine pancreatic insufficiency, inflammatory bowel disease, liver dysfunction affecting bile acid synthesis, biliary obstruction, celiac disease, short bowel syndrome, small intestinal bacterial overgrowth, hypobetalipoproteinemia, chylomicron retention disease, Whipple disease, intestinal lymphangiectasia, parasitic infection, and certain iatrogenic causes such as radiation or drug therapy [15,16].

Fat malabsorption can be investigated by analyzing dietary fat digestion, absorption, and—and in some cases—its intracellular lipid processing, using the experimental models illustrated in Figure 1. Section 4 further elaborates on model selection, emphasizing that the ideal choice depends primarily on the required sensitivity, physiological relevance, and depth of mechanistic insight.

Given the central role of the intestine in lipid metabolism and its implications for health and disease, a variety of experimental systems have been developed to investigate dietary fat absorption. This review aims to summarize and critically evaluate these models, emphasizing their respective strengths, limitations, and applications, and providing recommendations for selecting the most appropriate systems for each stage of dietary fat absorption.

## 2. Methods

To identify relevant experimental approaches, a comprehensive literature search was conducted using PubMed. The search focused on studies describing experimental models of dietary fat absorption using the keywords intestinal lipid absorption, dietary fat digestion, chylomicron assay, and intestinal lipoprotein transport. Given the large number of publications retrieved, not all techniques, methods, and models could be included in this review; this selective coverage, which is based primarily on how commonly they are utilized, should, therefore, be recognized as a limitation.

The collected studies encompass a wide range of experimental techniques, from animal models to organ-based systems, each designed to address different aspects of the absorption process. The following section categorizes and discusses these models in detail.

## 3. Experimental Models

Studying dietary fat absorption presents several methodological challenges. Unlike most cell types, enterocytes have an exceptionally short lifespan—approximately 3.5 days in humans and less than 3 days in laboratory rodents [17]. Consequently, the use of primary enterocytes is extremely limited [18]. These cells lose viability shortly after isolation, suggesting that they require specialized conditions to survive and function ex vivo.

Another major challenge arises from the route of lipid transport. Most lipoproteins secreted by enterocytes, namely chylomicrons and VLDLs, first enter the lymphatic circulation before reaching the systemic bloodstream [3]. This makes it difficult to collect their intact unmetabolized forms. Even when intestinal lymph samples are obtained, complete recovery of these lipoproteins is nearly impossible because some VLDLs—particularly the smaller ones [4]—may bypass the lymphatic system and enter the hepatic portal vein directly [9,10].

To overcome these challenges, researchers employ three broad categories of models:

1. In vivo, which best replicate physiological conditions
2. Ex vivo, which enable controlled studies of intact intestinal segments
3. In vitro, which offer high experimental control for mechanistic studies

Each category has unique advantages and limitations. The following sections examine these models in order of increasing experimental simplicity but decreasing physiological complexity.

### 3.1. In Vivo Models

In vivo models remain the cornerstone of intestinal lipid absorption research because they preserve the integrated physiology of the whole organism. Despite their advantages, they often suffer from incomplete recovery of secreted lipoproteins and the confounding influence of hepatic VLDL production [7,19,20,21,22].

Rodents remain the most widely used animals in dietary fat absorption research. Nonetheless, other species—including swine [23,24], felines [25], canines [26], primates [27], birds [28], and fish [29]—have also been employed. All these animals, including fish [30], are capable of producing intestinal lipoproteins. However, certain aspects of their gastrointestinal physiology differ from that of humans. For example, rats lack a gallbladder, and the livers of mice and rats can produce ApoB48-containing lipoproteins [31]. Such interspecies differences should be carefully considered when selecting and interpreting animal models.

Within in vivo systems, several experimental methods are available, each designed to address specific aspects of dietary fat absorption.

#### 3.1.1. Lymph Duct Cannulation

Regarded by many basic science researchers as the gold standard for studying intestinal lipid transport, the lymph fistula model was first described by Bollman et al. in their pioneering report on mesenteric and thoracic lymph duct cannulation [32]. As most intestinal lipoproteins are collected by the mesenteric lymph duct and subsequently drained into the thoracic duct before entering the venous circulation via the subclavian vein [3], cannulation of either duct enables the collection of intestinal lipoproteins prior to their entry into systemic blood.

Approximately five decades later, refinements to the model incorporated duodenal and jugular vein cannulations for rehydration and blood sampling, respectively [33]. To preserve physiological lymph flow, subsequent modifications allowed the procedure to be performed in conscious rodents [34]. For a detailed discussion of this conscious lymph fistula model, the paper by Ko et al. from Patrick Tso’s laboratory provides an excellent reference [35]. More recently, the surgical procedure streamlined from a two-day to a one-day protocol, significantly improving animal survival rates [36]. A video demonstration has also been published to facilitate the visualization of this technique [37].

The lymph fistula model remains a robust and informative approach for several reasons. First, it enables the isolation of intestinal lipoproteins that have not undergone significant metabolism, thus providing direct insight into the transport phase of dietary fat absorption. Except for the smaller VLDLs, most ApoB-containing intestinal lipoproteins enter the lymph before reaching the circulation. Second, continuous lymph collection is possible if animals are adequately hydrated—typically by an intraduodenal infusion of lipid emulsions—allowing for kinetic analyses of lipid absorption and secretion. Third, intestinal lipoproteins are highly concentrated in the lymph, eliminating the need for ultracentrifugation, though the process can still be used to separate chylomicrons from VLDLs. When combined with isotopic labeling techniques (see Section 3.1.3), this model can also yield valuable data on lipid uptake and intracellular processing within enterocytes.

Despite its strengths, the lymph fistula model presents significant technical challenges. Complete recovery of all absorbed dietary lipids is rarely achievable, as smaller VLDLs may bypass the lymphatic system by entering the portal vein, typically resulting in only about 55–75% recovery of the administered fatty acid dose [7,21,22]. This variability is particularly pronounced in female animals, whose lymphatic triglyceride transport is more inconsistent due to hormonal regulation. These observations argue for the need of complementary approaches to assess the portal transport, which is discussed in the following section.

#### 3.1.2. Portal Vein Cannulation

Portal vein cannulation allows direct sampling of lipoproteins entering the liver from the intestine. Studies employing isotope labeling in male rats equipped with duodenum, carotid artery, and portal vein cannulas have demonstrated that approximately 39% of dietary triglycerides are transported via the portal vein [10], whereas about 54% are transported through the lymphatic system [38].

These findings indicate that although the lymphatic route represents the predominant pathway for dietary fat transport, the contribution of the portal route cannot be overlooked. Owing to the tighter endothelial junctions of blood capillaries [4], most chylomicrons and large VLDLs are presumed to enter the lymphatic circulation. This interpretation is supported by evidence showing that only about 1–2% of lymph-derived lipoproteins have diameters ≤ 20 nm [39], and that the majority of isotope-labeled lipids recovered from portal blood reside in the higher density fraction (d > 1.006 g/mL) [10].

Beyond these structural factors, biological sex adds a regulation dimension to lipid transport. Male mice, which typically consume more dietary fat, exhibit greater chylomicron production, and have been observed to generate larger intestinal lipoproteins than females—even when both are administered equivalent lipid loads [8]. Hormonal influences appear central to this difference: testosterone increases lipoprotein size but decreases particle number; estrogen slightly increases particle number without affecting size; and progesterone exerts intermediate effects, modestly increasing lipoprotein size without altering particle number. Consequently, female mice—particularly during certain phases of estrous cycle—exhibit reduced lymphatic lipid transport, likely because their smaller intestinal lipoproteins preferentially enter the portal circulation rather than the lymphatic system [7]. Additionally, estrogen-mediated activation of vascular endothelial growth factor A (VEGF-A) signaling can further limit lipoprotein entry into intestinal lymphatics.

Together, these findings highlight the importance of sex-dependent regulation of intestinal lipoprotein size as a determinant of lipid transport pathway selection. Further research is warranted to elucidate how dietary fat load and sex hormones jointly shape the partitioning of lipid transport between the portal and lymphatic routes, and how this, in turn, influences systemic lipid metabolism and metabolic health.

#### 3.1.3. Isotope Labeling

As the previous sections illustrate, distinguishing between lipid transport routes require precise tracking. Isotope labeling provides this capability, enabling researchers to trace the metabolic fate of dietary lipids using radiolabeled (^3^H, ^14^C) or stable (^13^C) isotopes. To ensure accurate results, labeled lipids should be thoroughly mixed with unlabeled lipids prior to administration, which is usually by oral gavage or intraduodenal infusion. Unlike radioactive tracers, which are quantified using a scintillation counter, ^13^C-labeled samples require analysis by mass spectrometry.

For triglyceride tracing, isotope labeling is typically applied to the fatty acid moieties rather than the glycerol backbone because the primary goal is to track the bulk of the absorbed fat [7,21,22]. In contrast, during cholesteryl ester feeding experiments, the cholesterol backbone is usually labeled instead of the fatty acid, as investigators are mainly interested in monitoring cholesterol uptake and metabolism. The specific labeling position is also crucial when analyzing lipid composition, as appropriate labeling enables more accurate detection of lipid re-esterification abnormalities within intestinal tissues [22].

#### 3.1.4. Fecal Analysis

To assess lipid uptake efficiency, fecal isotope assays provide a complementary endpoint to lymph or portal measurements. The fecal dual-isotope method, for example, uses cholesterol and sitosterol tracers to calculate cholesterol absorption [22]. In this approach, animals are gavaged with a lipid mixture containing isotope-labeled cholesterol and sitosterol. Because sitosterol, a plant sterol, is minimally absorbed, cholesterol uptake can be calculated from the fecal ratio of labeled cholesterol to sitosterol. The fecal dual-isotope method is well suited for assessing intestinal cholesterol uptake over approximately 24 h. For shorter experimental periods (e.g., 6 h), a continuous intraduodenal infusion of a lipid mixture containing isotope-labeled cholesterol may be used. This technique, however, requires duodenal cannulation and the complete collection of luminal contents at the end of the experiment. Since these two approaches differ in sensitivity and physiological relevance, data interpretation must be performed accordingly. Based on our experience, the fecal dual-isotope method provides more physiological results but is less sensitive [22].

For examining fatty acid uptake, the triolein/triether fecal dual-isotope method can be applied [40]. The principal is analogous to that of the cholesterol/sitosterol assay described above. Alternatively, a continuous intraduodenal infusion of isotope-labeled fatty acids can be performed for shorter study durations, followed by quantification of fatty acid recovery from the luminal content [22].

A third approach is the sucrose polybehenate method [41], a component of the fat substitute olestra, serves as a non-absorbable marker. In this method, animals are fed a prepared diet containing absorbable fatty acids and sucrose polybehenate, and fecal pellets are collected after two nights of feeding. Dietary fat absorption is then determined from the fecal ratio of absorbable fatty acids to behenic. Unlike the dual-isotope methods, this approach does not require isotopes and relies on gas chromatography for analysis. Complete fecal collection is not essential for accuracy. Similar to the dual-isotope methods, the sucrose polybehenate and triolein/triether approaches are considered more physiological but less sensitive than the continuous intraduodenal infusion technique [22].

#### 3.1.5. Analysis of Peripheral Blood

The lipid and apolipoprotein concentrations in the chylomicron and VLDL fractions isolated from systemic blood can provide insight into dietary fat transport. However, as noted above, these circulating lipoproteins have undergone greater metabolic modification compared to those recovered directly from lymph samples. To minimize post-secretory metabolism, the non-ionic detergent Triton WR1339 can be administered, as it prevents the hydrolysis of triglycerides within chylomicrons and VLDLs [42].

Distinguishing intestinal VLDLs from hepatic VLDLs in blood samples remains a major challenge. This difficulty is compounded by the fact that, unlike in humans, rodent hepatic VLDLs may also contain ApoB48 [31], further complicating data interpretation derived from systemic circulation.

#### 3.1.6. Analysis of Intestinal Tissues

Intestinal tissue serves as an informative sample for elucidating both molecular pathways and morphological alterations associated with lipid absorption. Quantification of mRNA and protein expression is commonly employed to assess the regulation of lipid transporters and metabolic enzymes. In parallel, mucosal lipid composition, using thin-layer chromatography, enables the detection of abnormalities in lipid re-esterification and lipoprotein assembly [22].

The ultrastructure of the small intestine can also be examined using transmission electron microscopy, providing a non-biochemical approach to study dietary fat absorption. For example, Takahara et al. demonstrated, through regional analysis of intestinal lipoprotein size distribution, that smaller lipoproteins preferentially enter the portal venous system, whereas larger particles are transported via the lymphatic system [11]. Although earlier studies using portal vein cannulation produced similar conclusions [9,10], transmission electron microscopy offered additional spatial insights by revealing mucosal regions where large intestinal lipoproteins accumulate.

As noted above, prior to the intestinal tissue collection, fasted animals are typically administered a defined amount of dietary lipids, either by oral gavage or intraduodenal infusion. Among these, oral gavage is less invasive and more physiological than intraduodenal infusion.

Table 1 summarizes the major biological samples that can be collected and analyzed in in vivo studies of dietary fat absorption, along with their analytical applications, research purposes, and key limitations.

#### 3.1.7. The Utility of Pharmacological Agents

Pharmacological agents provide yet another means of dissecting lipid absorption mechanisms. ApoB inhibitors (e.g., mipomersen) and microsomal triglyceride transfer protein inhibitors (e.g., lomitapide), both clinically used for treating homozygous familial hypercholesterolemia, can be employed experimentally to modulate ApoB expression and microsomal triglyceride transfer protein activity, respectively [43]. Ezetimibe, an inhibitor of intestinal cholesterol absorption, is useful for manipulating enterocyte cholesterol uptake, while orlistat, a gastric and pancreatic lipase inhibitor, prevents the hydrolysis of dietary triglycerides [44]. In addition, the increasingly popular glucagon-like peptide-1 (GLP-1) receptor agonists (e.g., semaglutide), widely prescribed for weight management and type 2 diabetes, can be applied to delay gastric emptying, thereby influencing the kinetics of lipid delivery to the intestine [44]. Collectively, these agents offer valuable experimental tools for probing various aspects of dietary fat digestion, absorption, and transport.

While in vivo models offer the most physiologically integrated insights, their technical complexity and limited throughput have prompted the development of ex vivo systems, which balance physiological relevance with experimental control.

### 3.2. Ex Vivo Models

Ex vivo models employ isolated segments of the small intestine, most commonly the jejunum, to study lipid absorption under controlled conditions. Although the duodenum also plays a major role in dietary fat absorption, it is less frequently used in ex vivo studies, likely due to its shorter length compared with the jejunum. In the following section, we discuss the two principal ex vivo models employed in the study of dietary fat absorption: the everted gut sac model and the human fetal gut model.

#### 3.2.1. Everted Gut Sac

The everted gut sac model was introduced by Wilson and Wiseman in 1954 to investigate the transport of substances from the mucosal to the serosal layer of the small intestine [45]. Using this model, they successfully demonstrated the movement of glucose and methionine against their concentration gradient. The method involved using segments of the small intestine from rats or golden hamsters as an ex vivo system to study intestinal transport [45]. Since its introduction, the everted gut sac model has been applied to a wide range of animal species, including pigs, frogs, hamsters, catfish, rabbits, chickens, guinea pigs, rats, sheep, goldfish, and mice [46]. Among these, rats and mice are the most commonly used due to their availability, small size, and well-characterized physiology. Over the past several decades, the model has undergone various modifications to improve intestinal tissue viability, and it remains one of the most widely utilized ex vivo models for investigating dietary fat absorption.

The small intestine is the primary site of nutrient absorption in humans [47]. It measures approximately 6–7 m in length and 2.5–3 cm in diameter, compared to the large intestine, which is about 1.5 m long and 6–7.5 cm in diameter [48]. Despite its narrower lumen, the small intestine possesses a much larger absorptive surface area due to extensive structural adaptations. Specifically, the presence of plicae circulares (circular folds), villi, and microvilli amplifies its surface area by 30–600 fold [48]. The duodenum and jejunum are particularly enriched with circular folds [49], which are themselves lined with numerous villi—finger-like projections that facilitate nutrient absorption. Each villus is composed of many enterocytes, whose apical microvilli form the brush border membrane, further maximizing the absorptive surface.

**Figure 2:**
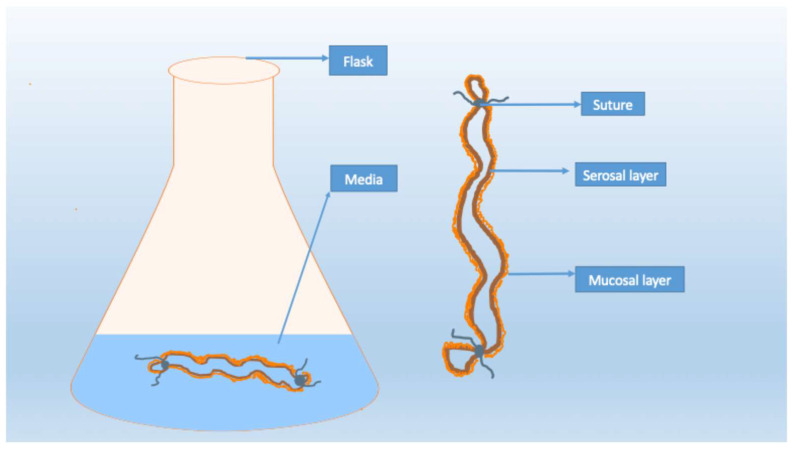
The everted gut sac model. An intestinal segment is everted so that the mucosal layer faces outward and the serosal layer encloses the inner compartment. The ends are tied with sutures; the sac is filled with a proper solution and then placed in a flask containing incubation medium at 37 °C.

To establish an everted gut sac, a small segment of the duodenum, jejunum, or ileum (typically 4–6 cm long) is excised. After washing it with an ice-cold physiological solution, the segment is carefully everted using a glass rod. One end is tied with a silk braided suture and filled with an appropriate solution, then sealed by tying the opposite end with another suture. As illustrated in Figure 2, the filled intestinal sac is placed in a flask containing incubation medium and maintained at 37 °C [46].

Several studies have utilized the everted gut sac model to elucidate mechanisms of dietary fat absorption. The formation of chylomicrons within the everted gut sac has been confirmed by analyzing the serosal fluid using transmission electron microscopy [50]. However, exposure of the everted jejunum to low concentrations of calcium and magnesium markedly reduced chylomicrons formation, while physiological concentrations of these ions restored chylomicron formation [51]. The model has also been employed to compare the effects of free bile acids and conjugated bile salts on fatty acid esterification and triglyceride synthesis in the intestine [52]. These studies demonstrated that conjugated bile salts more effectively promoted fatty acid uptake and triglyceride re-esterification—key upstream steps in lipoprotein formation. Likewise, other investigations have explored the influence of additional bile components on lipid absorption using this model [53,54,55].

The everted gut sac offers several advantages as an ex vivo tool. It provides a relatively accurate representation of intestinal physiological conditions, allowing for efficient nutrient uptake due to the large absorptive surface area [46]. This technique is also simple to prepare and handle, cost-effective and time-efficient compared to other models [46].

Nevertheless, the model has several limitations. The intestinal tissue exhibits short viability, typically restricting experimental duration to a few hours or days [56]. To extend tissue viability, some researchers have proposed using cell culture media instead of standard buffer solution. Additionally, the sac is fragile and susceptible to mechanical damage during preparation or incubation. Its functionality may also vary depending on multiple factors, including animal-related variables (e.g., age, sex, species, disease state, and chronic treatment), experimental conditions (e.g., pH, aeration, temperature, and substrate concentration), and anatomical origin (e.g., duodenum vs. jejunum vs. ileum) [46]. Moreover, because the everted gut sac utilizes only a short intestinal segment, it produces fewer intestinal lipoproteins than the lymph fistula model, making the isolation of intact lipoproteins more challenging.

Although the everted gut sac model is most commonly prepared from adult animal intestines, it can also be adapted for use with human fetal gut tissue [57]. As discussed in the following section, the human fetal gut model has been successfully employed to study dietary fat absorption and offers unique insights into lipid processing during development.

#### 3.2.2. Human Fetal Gut

Human fetal gut tissue is typically obtained from patients undergoing pregnancy termination, and its use in research requires special ethical and institutional approval. Although the explants generally remain viable for several days in culture, morphological alterations of the epithelial layer have been reported after approximately 24 h [58]. For these reasons, the human fetal gut model has not been as widely used as other intestinal models. Nonetheless, it provides a unique experimental system for investigating human intestinal development and the ontogeny of dietary fat absorption.

The jejunum is the most commonly isolated segment for this model. When obtained from 16- to 20-week human fetuses, the jejunum can be identified as the proximal half of the small intestine, excluding the first 3 cm from the duodenum [59]. Explants measuring approximately 3 × 7 cm can be excised from the isolated jejunal tissue. Importantly, this model has been demonstrated to express microsomal triglyceride transfer protein [60], synthesize more ApoB48 preferentially over ApoB100 [59], and produce intestinal lipoproteins [61]. Both chylomicrons and VLDLs can be isolated from the jejunal explant homogenate and culture media through sequential ultracentrifugation, confirming the presence of functional lipoprotein assembly machinery in the developing human intestine.

While ex vivo models bridge the gap between organismal and cellular systems, advances in cell culture have given rise to powerful in vitro models, which allow precise mechanistic interrogation of enterocyte lipid metabolism under controlled conditions.

### 3.3. In Vitro Models

In vitro systems provide exceptional experimental control and scalability, making them invaluable for mechanistic studies. Except for more complex gut-on-a-chip systems, the in vitro models described below are also more cost-effective than the in vivo models discussed previously. Additionally, in vitro systems enable complete recovery of intestinally produced lipoproteins, which is not possible in the in vivo systems.

However, in vitro models also present notable limitations. They lack the physiological complexity and multi-organ interactions inherent in living systems, and cellular conditions may be altered artificially by the culture environment. As a result, their findings may not fully reflect human physiological relevance, underscoring the importance of interpreting in vitro data within the broader context of in vivo and ex vivo research. The following sections describe commonly used enterocyte-based in vitro models, including Caco-2 cells, IPEC-1 cells, and enteroids, along with emerging gut-on-a-chip platforms.

#### 3.3.1. Caco-2 Cells

Caco-2 (Cancer Coli-2) cells are among the most widely used in vitro models for studying dietary fat absorption, largely due to their commercial availability and extensive characterization. However, as discussed below, they are also technically more challenging to work with than many other cell lines.

Although Caco-2 cells originate from human colon adenocarcinoma, upon spontaneous differentiation they acquire biochemical and morphological characteristics similar to those of small intestinal enterocytes [62]. One of their most distinctive features is the ability of fully differentiated Caco-2 cells to synthesize chylomicrons—a property normally restricted to enterocytes during the postprandial state [21,22,63]. In contrast, VLDLs are synthesized by both the hepatocytes and enterocytes. This unique ability to produce chylomicrons underlies the widespread use of Caco-2 cells as a model system for intestinal lipid absorption.

Multiple studies have demonstrated lipoprotein production by Caco-2 cells using biochemical and ultracentrifugation analyses [64,65,66,67]. Although ultracentrifugation allows isolation of lipoprotein fractions, this method can overestimate chylomicron formation due to the aggregation or clustering of lipoproteins during processing [68]. Therefore, the presence and morphology of chylomicrons are best confirmed by transmission electron microscopy in conjunction with biochemical assays.

In our studies, a mixture of oleic acid, lecithin, and bile salt provided the optimal stimulus for fully differentiated Caco-2 cells to produce chylomicrons [8,68]. Given the biochemical similarity between chylomicrons and VLDLs, we confirmed chylomicron formation both biochemically and by transmission electron microscopy. Because chylomicrons are the largest lipoproteins, they possess the highest triglyceride-to-ApoB ratio among all lipoproteins—a feature that can be used to estimate their relative size [8,68]. Our model does not require isotope-labeled fatty acids and is capable of producing ApoB48-containing lipoproteins, consistent with intestinal origin.

To more closely mimic physiological transport, a semipermeable membrane system should be employed, allowing simultaneous lipid incubation in the apical compartment and lipoprotein collection from the basolateral compartment. For lipoprotein particle size analysis, we recommend using 75 mm semipermeable inserts, which accommodate more cells and thus yield a greater number of lipoproteins. Prior to transmission electron microscopy, the collected lipoproteins should be concentrated via gradient ultracentrifugation to ensure clear particle visualization.

Although only about 21% of lipoproteins produced by our Caco-2 model are chylomicrons, compared to approximately 30% and 67% in the female and male mouse lymph fistula models [8], respectively, this discrepancy is not unexpected. Many VLDL particles, particularly smaller ones, do not enter the lymphatic system. As indicated in Section 3.1.2, roughly 39% of dietary triglycerides are absorbed directly into the portal vein [10]. Because VLDLs have a lower triglyceride-carrying capacity than chylomicrons, a higher particle number is required to transport equivalent triglyceride quantities. Thus, the apparent difference in chylomicron-to-VLDL ratios between our Caco-2 and lymph fistula models may be less substantial than it initially appears.

Genetic manipulation of Caco-2 cells presents additional challenges. These cells typically exhibit 30–60% transfection efficiency, and transgene expression is often transient, limiting their usefulness in dietary fat absorption studies, since only fully differentiated cells can effectively produce chylomicrons [69]. Achieving stable transfection is particularly difficult, likely due to efflux of selection antibiotics and the interference of these antibiotics with efflux transporter activity [70]. Consequently, lentivirus transduction has been proposed as a more effective approach for achieving stable gene expression in Caco-2 cells [69]. Indeed, lentivirus-transduced Caco-2 cells can maintain expression of the gene of interest for at least 12 passages.

In addition to Caco-2 cells, the only other cell line known to produce chylomicrons is the intestinal porcine epithelial cells (IPEC-1). However, IPEC-1 cells must overexpress ApoA-IV to synthesize chylomicrons, limiting their routine use for this purpose.

#### 3.3.2. IPEC-1 Cells

IPEC-1 cells are spontaneously immortalized epithelial cells derived from the neonatal porcine distal small intestine. Upon differentiation, they develop apical microvilli and express both ApoB100 and ApoB48, reflecting enterocyte-like characteristics [71].

To induce chylomicron production, IPEC-1 cells require overexpression of ApoA-IV. In the absence of ApoA-IV overexpression, lipid-treated cells secrete mostly VLDL-sized lipoproteins, with only about 1% classified as chylomicrons [72]. The average diameter of the intestinal lipoproteins under these conditions is approximately 54.3 nm. When ApoA-IV is overexpressed and lipids are supplied, the mean lipoprotein diameter increases to 87.0 nm, consistent with chylomicron formation.

For comparison, lymph fistula studies in mice have reported average intestinal lipoprotein diameters of 83.35 nm in females and 128.41 nm in males when challenged with dietary lipids [8]. Given that in vitro systems allow isolation of smaller VLDLs, lipoproteins from IPEC-1 cultures are expected to exhibit smaller mean diameter than those derived from in vivo models. Typically, ApoB-containing intestinal lipoproteins measuring <80 nm are classified as VLDLs, whereas those ≥80 nm are considered chylomicrons.

ApoA-IV promotes chylomicron production in IPEC-1 cells primarily by enhancing the incorporation of triglycerides into nascent lipoprotein cores, a process mediated by the upregulation of microsomal triglyceride transfer protein [73,74]. The residues 344–354 of human ApoA-IV play a key role in facilitating this activity, while its *C*-terminal EQQQ repeat domain appears to exert inhibitory effects. Accordingly, IPEC-1 cells overexpressing a truncated form of human ApoA-IV lacking the EQQQ-rich *C*-terminus produce extremely large lipoproteins, estimated to be 27-fold larger than normal [72]. Because of their size, these particles could not be visualized by negative staining and instead required imaging via the osmium vapor technique under transmission electron microscopy.

All the cell lines discussed above are typically cultured in two-dimensional monolayer system. More physiologically relevant three-dimensional models, such as enteroids and gut-on-a-chip systems, will be discussed in the following sections.

#### 3.3.3. Enteroids

Enteroids are three-dimensional epithelial cultures derived from intestinal crypt stem cells isolated from human or animal tissue and subsequently grown in solubilized basement membrane matrices such as Matrigel [75,76]. The crypt cells can be obtained from the duodenum, jejunum, or ileum of adult intestines, though those isolated from the proximal third of the small intestine are generally more effective at producing chylomicrons [77]. Within the matrix, these intestinal stem cells can be induced to differentiate into the major epithelial cell types of the intestine, including enterocytes, enteroendocrine cells, goblet cells, and Paneth cells. Alternatively, the undifferentiated crypts can also be cryopreserved for later use.

Mature enteroids exhibit a characteristic three-dimensional hallow spherical morphology, in which the apical membrane of the cells faces inward toward the central lumen, while the basolateral membrane faces outward toward the extracellular matrix. Because of this orientation, the apical and basolateral compartments are not physically separated. Consequently, simultaneous lipid incubation at the apical surface and lipoprotein collection from the basolateral side—as is possible in Caco-2 cells grown on semipermeable membrane systems—cannot be achieved. Whether this limitation significantly impacts experimental outcomes depends largely on the specific research objectives, since the cells remain structurally polarized despite the absence of physical compartmentalization.

Enteroids have been effectively used to investigate dietary fat absorption and lipoprotein synthesis [77,78]. To stimulate chylomicron formation, mature enteroids are typically incubated for two hours with oleic acid complexed to albumin, followed by replacement with fresh medium for an additional four hours. Lipoprotein secreted into the culture medium are then isolated by ultracentrifugation.

When challenged with the oleic acid-albumin complex, the average diameter of intestinal lipoproteins secreted by enteroids is approximately 125 nm, compared to about 100 nm in unchallenged controls. When stimulated with oleic acid-containing mixed micelles, the average diameter only increases modestly to around 110 nm [77]. For comparison, lymph fistula studies have reported average intestinal lipoprotein diameters of 63.16 nm and 79.74 nm in preprandial female and male mice, respectively, and 83.35 nm and 128.41 nm in postprandial female and male mice, respectively [8]. Therefore, the lipoproteins secreted by enteroids, particularly under basal conditions, appear larger than those observed in in vivo lymphatic studies, even when accounting for the exclusion of smaller VLDL particles in the latter.

Table 2 compares the average diameters of intestinal lipoproteins produced across the models discussed.

The enteroid model offers several advantages. It can be established using intestinal crypts from virtually any donor, including humans, and exhibits robust chylomicron production compared with immortalized cell lines such as Caco-2 and IPEC-1, which typically generate fewer chylomicrons. However, like most in vitro systems, enteroids lack intestinal motility and luminal fluid flow, factors that influence lipid digestion and absorption in vivo [79]. Additionally, the absence of a physical separation of apical and basolateral compartments limits the ability to use distinct incubation and collection media simultaneously, representing a significant methodological constraint for certain experimental designs.

#### 3.3.4. Gut-on-a-Chip

**Figure 3:**
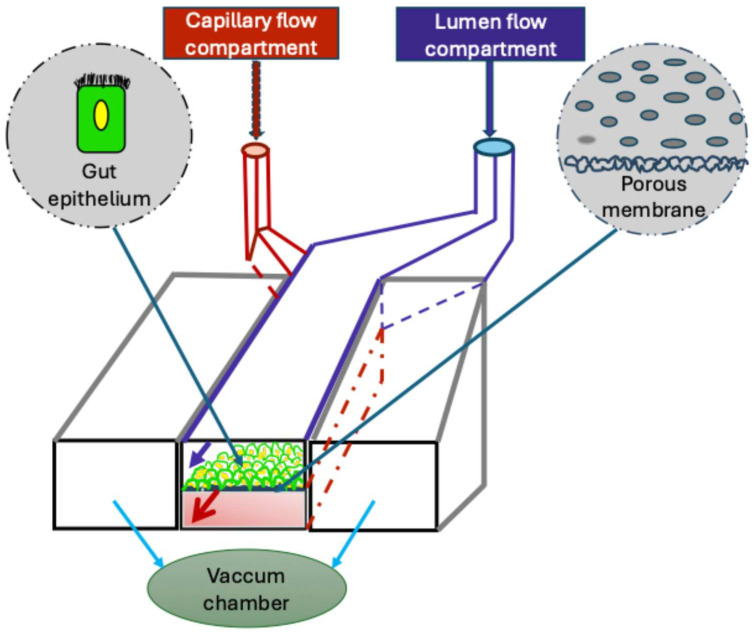
Gut-on-a-chip model. A typical gut-on-a-chip model features a porous membrane that separates the lumen flow compartment from the capillary flow compartment. The gut epithelium is cultured on the membrane surface facing the lumen compartment, while both compartments are continuously perfused to reproduce physiological shear stress. The entire device is enclosed within a vacuum chamber that induces cyclic mechanical deformation, simulating the peristaltic motions of the intestine.

As shown in Figure 3, a typical organ-on-a-chip model consists of a transparent polymer membrane that divides the chip into two microfluidic compartments. Each compartment, usually seeded with one or more cell types, is continuously perfused with a fluid flow that mimics physiological shear stress [79]. A variety of organ-on-a-chip systems have been developed, including models of the blood–brain barrier [80], heart [81,82], lung [83,84], kidney [85], bone marrow [86], liver [87], and intestine [79]. Because each model is engineered to recapitulate key structural and functional features of its respective organ, these systems provide powerful platforms to study organ-specific physiology, disease mechanisms, and therapeutic responses. For instance, the lung-on-a-chip has been used to investigate pulmonary thrombosis [83] and pulmonary edema [84], while the heart-on-a-chip enables studies of cardiac morphometrics, electrophysiology [81], and ischemia [82]. Similarly, tumor-on-a-chip facilitate the exploration of cancer pathogenesis and drug responses [88], and multiple organ systems have been designed to examine the inter-organ communication, such as between intestine and liver [89].

Several gut-on-a-chip systems have been successfully developed. One notable model, known as Gut Chip, was engineered to study the host-microbiome interaction [79]. This platform incorporates key physiological features such as peristalsis-like motion, a vascular microenvironment, crypt-villus architecture, and a resident microbiome. Within this model, Caco-2 cells differentiate into absorptive enterocytes, goblet cells, enteroendocrine cells, and Paneth cells, forming a polarized epithelial barrier with an overlying mucus layer. The mechanical deformations mimicking peristalsis help prevent bacteria overgrowth. While it remains to be determined whether this system can produce substantial quantities of chylomicrons upon lipid challenge, such capability would make it highly suitable for investigating how gut microbes modulate dietary fat absorption.

An alternative Intestine Chip model replaces Caco-2 with patient-derived enteroids, effectively combining the strengths of enteroid and gut-on-a-chip technologies [90]. This hybrid system enables personalized medicine approaches, allowing the study of patient-specific intestinal physiology and responses to treatment.

Another variant, the Digestion-on-a-chip model, employs three interconnected compartments representing the mouth, stomach, and intestine [91]. Although this system simulates sequential digestive processes and achieves faster milk protein hydrolysis than conventional batch digestion, its physiological digestion rate has not been fully optimized. While bile and pancreatic lipase are incorporated to simulate intestinal digestion, the digestion and absorption of dietary lipids have yet to be systematically characterized. Future refinements could adapt this platform for dietary fat digestion and absorption studies.

The NutriChip model is specifically designed to examine the effects of nutritional compounds on postprandial inflammation [92]. It co-cultures immune and intestinal epithelial cells and integrates an optical detection system for real-time, non-invasive quantification of interleukin-6 secretion. By screening various food products, the model helps identify potential anti-inflammatory nutrients. With further modification, the NutriChip could be adapted to monitor lipoprotein secretion in real time, providing a valuable tool for studying dietary fat absorption.

Overall, gut-on-a-chip technologies represent an exciting and rapidly evolving frontier in intestinal research. Although current systems have not yet been optimized for detailed studies of dietary fat absorption, ongoing advancements may soon enable the exploration of dynamic lipid metabolism, microbial interactions, and systemic physiological responses within a controlled microphysiological environment.

## 4. Integration of Experimental Models

**Figure 4:**
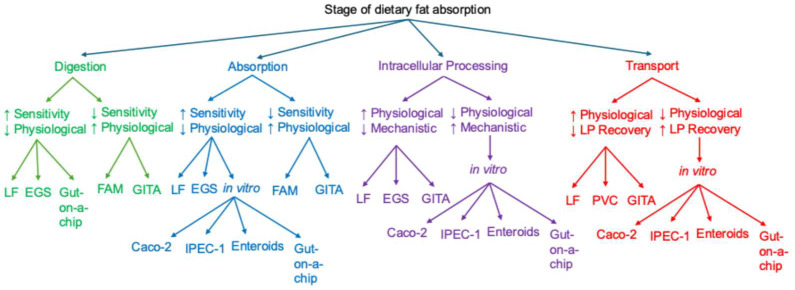
Guidelines for selecting experimental models to study dietary fat absorption. This flowchart outlines the recommended experimental models for investigating each stage of dietary fat absorption—digestion, absorption, intracellular processing, and transport—based on factors such as sensitivity, physiological relevance, mechanistic insight, and lipoprotein (LP) recovery (↑ = high, ↓ = low). For digestion studies using oral gavage followed by intestinal tissue analysis (GITA), labeled triglycerides should be employed instead of fatty acids, and complete luminal wash is essential for thin-layer chromatography analysis. Although GITA can also assess absorption, it is less ideal due to possible incomplete luminal wash recovery. The fecal analysis method (FAM) is generally preferred. When studying transport in female animals, the lymph fistula (LF) model is suboptimal due to variable lymphatic triglyceride transport, while the portal vein cannulation (PVC) model should be used exclusively for evaluating portal transport. The everted gut sac (EGS) model can effectively assess digestion, absorption, and intracellular processing, but is unsuitable for transport studies because of its limited LP recovery and inability to collect lymph or portal blood samples.

The study of dietary fat absorption is essential for elucidating mechanisms of lipid metabolism, understanding pathophysiology of metabolic syndrome, and addressing disorders of fat malabsorption. However, no single experimental model fully recapitulates the complexity of intestinal fat absorption, as each approach carries inherent methodological limitations. Future progress in this field will depend on integrative experimental strategies that combine these complementary models to overcome current limitations and bridge mechanistic insights with physiological relevance. Table 3 summarizes these models and their respective strengths, limitations, utilities, and validation strategies, while Figure 4 provides a practical guide for selecting the appropriate model to study specific stages of dietary fat absorption.

### 4.1. In Vivo Models: Unmatched Physiological Relevance, Limited Mechanistic Resolution

In vivo models, including lymph fistula, portal vein cannulation, fecal analysis, and gavage followed by intestinal tissue analysis, are indispensable for capturing the intact physiological context. Their strengths derive from their ability to preserve native anatomy, vascular and lymphatic flow, neuroendocrine signaling, and hormonal regulation.

#### 4.1.1. Transport Between Lymphatic and Portal Route

Lymph fistula model uniquely enables simultaneous assessment of digestion, absorption, intracellular processing, and lymphatic lipoprotein transport. Its lipoprotein recovery is typically 55–75%, with remainder likely entering the portal circulation. The model is, however, less reliable in female animals that produce more VLDLs and fewer chylomicrons due to their hormonal regulation—a critical consideration for experimental design. Portal vein cannulation model isolates the portal blood samples and yields about 39% recovery of fed fatty acids. This model is suitable only for studying portal transport, as it excludes larger lipoproteins.

#### 4.1.2. Digestion and Absorption

Fecal analysis method provides high physiological relevance for studying digestion and absorption but is less sensitive. Gavage of labeled triglycerides followed by intestinal tissue analysis, can serve as an alternative to fecal analysis method for studying digestion and absorption. However, both approaches are unable to recover lipoproteins, limiting their use for transport studies.

### 4.2. Ex Vivo Models: Preserved Tissue Structure with Limited Functional Scope

Ex vivo models, such as the everted gut sac and human fetal gut, provide intermediate levels of complexity. They retain epithelial architecture and brush-border function but lack systemic circulation and complete lipoprotein recovery. As such, they can be used to study digestion, absorption, and intracellular processing, but not transport. Their short viability restricts the experimental window. Unlike human fetal gut, which is difficult to obtain, everted gut sac is relatively easy to handle. Human fetal gut, however, is the ideal model to study ontogeny of fat absorption.

In general, ex vivo systems provide more mechanistic access than in vivo models but cannot capture systemic lipid trafficking.

### 4.3. In Vitro Models: Highest Mechanistic Resolution, Lowest Physiological Relevance

In vitro models, including Caco-2, IPEC-1, enteroids, and gut-on-a-chip technologies, enable precise dissection of molecular pathways governing lipid uptake, intracellular trafficking, and lipoprotein assembly. Caco-2 model is cost-effective, well characterized, and allows for complete lipoprotein recovery. However, it has less robust chylomicron output compared to other in vitro models. IPEC-1 model requires ApoA-IV overexpression for chylomicron production. Enteroids allow for donor-specific cells to be examined, preserving genetics, disease status, and interindividual variation. However, enteroids lack a physical apical-basolateral separation. Gut-on-a-chip model introduces microfluidic flow, shear stress, cyclic stretch, and compartmentalization, but it is not yet widely utilized for dietary fat absorption studies.

### 4.4. Future Directions: Integrating Systems to Bridge Physiological and Mechanistic Gaps

Recognizing the limitations of existing models, major advances in the field will rely on integrative experimental strategies that combine multiple platforms. One particularly promising direction involves combining enteroid and gut-on-a-chip technologies [90]. This integrative platform enables donor-derived intestinal cells to be studied within a microfluidic environment that more closely mimics in vivo physiology, including dynamic flow, shear stress, and compartmentalized luminal and basolateral interfaces—features not achievable in traditional enteroid cultures [77,78]. Moreover, when coupled with advanced omics approaches, such as transcriptomics and lipidomics, this system holds significant potential for identifying genes and pathways that regulate dietary fat absorption and lipoprotein assembly.

## 5. Conclusions

The progress of dietary fat absorption will likely come from thoughtfully combining in vivo, ex vivo, and/or in vitro models—each selected according to the specific biological question. Incorporating sex as a biological variable, particularly in transport models, will further strengthen study design and improve reproducibility. As emerging platforms such as enteroid-based microfluidic systems mature, the field stands poised to generate deeper, more physiologically grounded insights into how dietary lipids are absorbed, transported, and dysregulated in metabolic disease.

## Figures and Tables

**Table 1:** Summary of sample types, analytical methods, purposes, and limitations in in vivo studies of dietary fat absorption.

Sample	Analysis	Purpose	Limitations
Fecal matter	Measurement of fecal lipid content	Detect fat malabsorption	Provide little information on intestinal lipoprotein transport
Lymph	Lipid and apolipoprotein quantification (VLDL, chylomicron, or total fractions); electron microscopy	Analyze intestinal lipoprotein transport and particle size	Smaller intestinal VLDLs may be excluded
Peripheral blood	Lipid and apolipoprotein quantification from VLDL and chylomicron fractions	Provide indirect information on intestinal lipoprotein transport	Data affected by metabolic variability; difficult to distinguish intestinal from hepatic VLDLs
Portal blood	Lipid and apolipoprotein quantification from VLDL fraction	Assess transport of smaller intestinal VLDLs	Larger intestinal lipoproteins are excluded
Intestinal tissues	Microscopy; mRNA and protein expression analyses; mucosal lipid composition profiling	Detect morphological and molecular changes; assess intestinal lipoprotein size and lipid re-esterification	Require complementary data from other sample types to confirm findings
Other tissues (e.g., adipose, muscle)	Lipid quantification (often using isotope labeling)	Determine uptake of dietary lipids by peripheral tissues	Provide limited information on intestinal absorption and lipoprotein transport

**Table 2:** Comparison of intestinal lipoprotein sizes in different experimental models.

Model/Source	Condition or Treatment	Average Lipoprotein Diameter and Range (nm)	Approximate % VLDLs and % Chylomicrons [References]
Caco-2 cells	Control (not stimulated with lipid)	28 (mostly 10–40)	96% VLDLs and 4% chylomicrons [68]
Caco-2 cells	Fatty acid/lecithin/bile salt mixture	54 (mostly 10–140)	79% VLDLs and 21% chylomicrons [68]
IPEC-1 cells	Fatty acid-albumin complex without ApoA-IV overexpression	54.3 (mostly 30–80)	99% VLDLs and 1% chylomicrons [72]
IPEC-1 cells	Fatty acid-albumin complex with ApoA-IV overexpression	87.0 (mostly 30–180)	47% VLDLs and 53% chylomicrons [72]
Enteroids	Control (not stimulated with lipid)	100 (mostly 50–250)	38% VLDLs and 62% chylomicrons [77]
Enteroids	Fatty acid-albumin complex	125 (mostly 50–250)	15% VLDLs and 85% chylomicrons [77]
Enteroids	Oleic acid-containing mixed micelles	110 (mostly 50–175)	33% VLDLs and 67% chylomicrons [77]
Mouse lymph fistula	Preprandial female	63.16 (mostly 20–140)	86.58% VLDLs and 13.42% chylomicrons [8]
Mouse lymph fistula	Preprandial male	79.74 (mostly 40–180)	70.63% VLDLs and 29.37% chylomicrons [8]
Mouse lymph fistula	Postprandial female	83.35 (mostly 20–220)	69.37% VLDLs and 30.63% chylomicrons [8]
Mouse lymph fistula	Postprandial male	128.41 (mostly 40–260)	32.54% VLDLs and 67.46% chylomicrons [8]

**Table 3:** Summary of experimental models of dietary fat absorption, highlighting their strengths, limitations, utilities, and validation strategies.

Model	Strengths	Limitations	Utilities	Validation Strategies
Lymph fistula	Considered the gold standard due to its ability to study digestion, absorption, intracellular processing, and transport	Smaller intestinal VLDLs may get excluded; require extensive surgical procedure	High physiological relevance overall but may not be ideal for studying female animals	Recovery of fed fatty acid dose is typically 55–75% (the other 25–45% may bypass the lymph and enter the portal vein) [7,21,22]
Portal vein cannulation	Ideal for studying portal transport of dietary lipids	Larger intestinal lipoproteins get excluded; require extensive surgical procedure	Rarely used except for studying the physiology of portal transport	Recovery of fed fatty acid dose in portal vein should be around 39% [10]
Fecal analysis	Non-invasive and ideal for studying enterocyte uptake of dietary lipids (absorption)	Not generally used to study intracellular processing and transport	High physiological relevance for studying digestion and absorption	Less sensitive; 90% or more of fed fatty acid dose is absorbed [22,40,41]
Gavage + intestinal tissue analysis	No surgery involved; ability to provide morphological, molecular, and biochemical information	Require complementary data from other sample types to confirm findings	Provide some mechanical insights but isolation of intestinal lipoproteins is not possible	Complementary data from other sample types are often required; check for regurgitation [93]
Everted gut sac	Relatively easy to handle and less expensive	Short viability; not ideal for studying transport	Provide some mechanical insights; minimal isolation of intestinal lipoproteins	Tissue viability is a concern; experiments may need to be conducted within a day [50,51,52,53,54,55,56]
Human fetal gut	Ideal for studying the ontogeny of dietary fat absorption	Short viability; require special approval	Rarely used except for studying the development aspect of dietary fat absorption	Tissue viability is a concern; experiments may need to be conducted within a day [59,60,61]
Caco-2	Commercially available and well characterized; cost-effective; complete lipoprotein recovery	Lack physiological complexity; chylomicron production is less robust	Can be used for detailed mechanistic studies	Isolated lipoproteins should be confirmed biochemically and microscopically [8,68,69]
IPEC-1	Cost effective; complete lipoprotein recovery	Lack physiological complexity; require ApoA-IV overexpression to achieve robust chylomicron production	Can be used for detailed mechanistic studies	Isolated lipoproteins should be confirmed biochemically and microscopically [71,72,73,74]
Enteroids	Cells can be from any donor; robust chylomicron production; complete lipoprotein recovery	Lack physiological complexity; absence of physical separation between apical and basolateral compartments	Can be used for detailed mechanistic studies; allow donor cells to be used in the experiments	Isolated lipoproteins should be confirmed biochemically and microscopically [77,78]
Gut-on-a-chip	Can be designed to address a specific research question	Lack physiological complexity; not widely used yet for studying dietary fat absorption	Can be designed to study the mechanistic interaction of dietary fat absorption and other process	Isolated lipoproteins should be confirmed biochemically and microscopically [91,92]

## Data Availability

Not applicable.

## Abbreviations

The following abbreviations are used in this manuscript:

Caco-2	Cancer Coli-2
GLP-1	glucagon-like peptide-1
IPEC-1	intestinal porcine epithelial cells
VEGF-A	vascular endothelia growth factor A
VLDL	very low-density lipoprotein
